# Supplementary material for: Electrically tunable organic–inorganic hybrid polaritons with monolayer WS2
Source: Nat Commun. 2017 Jan 17;8:14097. doi: 10.1038/ncomms14097 (PMC5247603; doi:10.1038/ncomms14097)
Supplement: Supplementary Information — Supplementary Figures, Supplementary Notes and Supplementary References [file ncomms14097-s1.pdf]

Supplementary Information

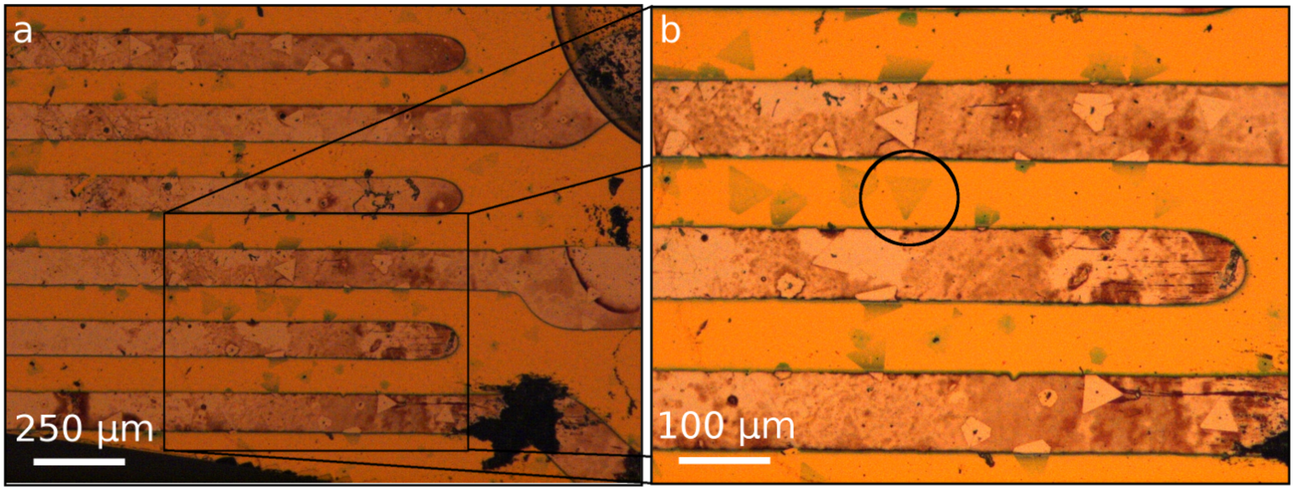

Supplementary Figure 1: **Optical microscope images of WS_2 flakes on DBR with electrodes.** a) Large field of view (x20) showing the individual silver electrodes, which are connected to either side. b) Zoom (x50) into region marked in a, showing randomly aligned WS_2 flakes on top of the silver electrodes. The circle marks one of the flakes used for this study.

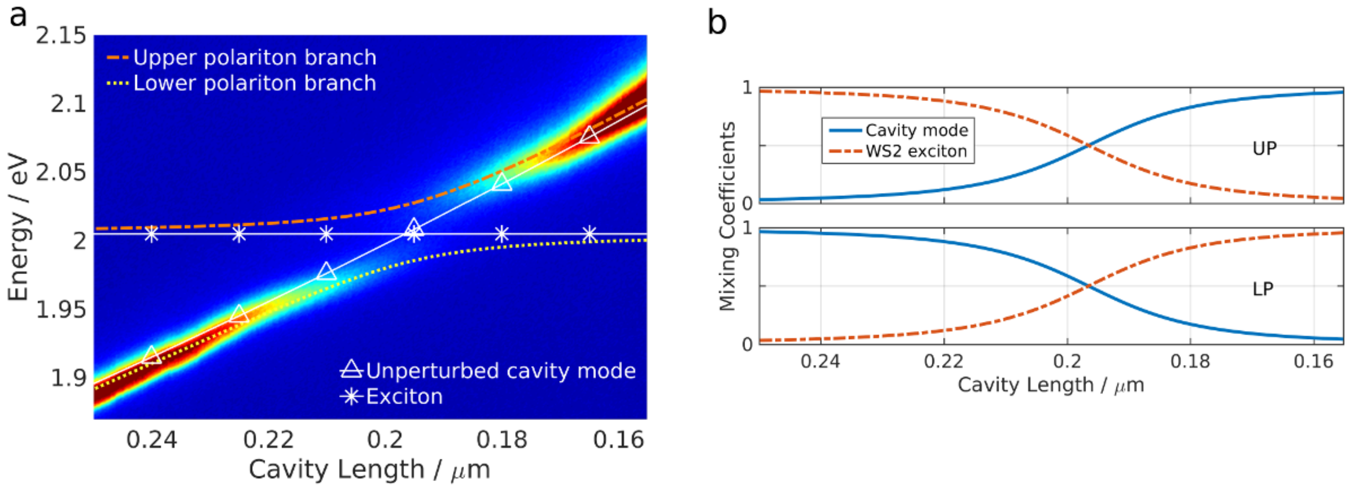

Supplementary Figure 2: **Polaritons with the inorganic component WS_2 only.** a) Successive transmission spectra as the cavity length is decreased from left to right. The unperturbed cavity mode (white continuous, Δ) has the longitudinal mode index $q = 3$. As it traverses the WS_2 exciton energy, the strong coupling is evidenced by the formation of lower (yellow, dashed) and upper (orange, dashed) polariton branches with a Rabi splitting of 70 meV. b) Photonic (blue, continuous) and excitonic (red, dashed) fractions of lower and upper polariton branch as the cavity length is swept.

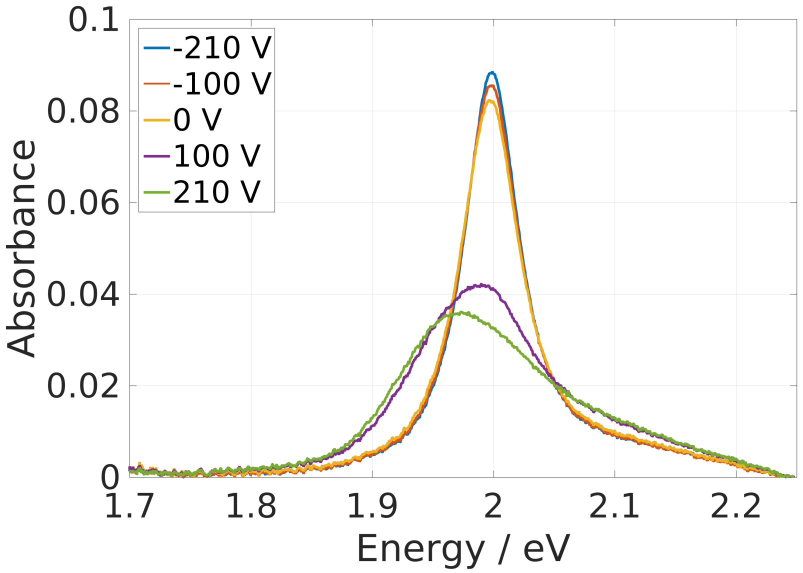

Supplementary Figure 3: **Electrically changed absorption profile.** WS_2 absorption profile as a function of the applied voltage. For large negative voltages the neutral exciton X^0 state dominates the profile (depletion of free electrons), while for the opposite bias the spectral weight shifts towards the charged exciton X^- (abundance of free electrons).

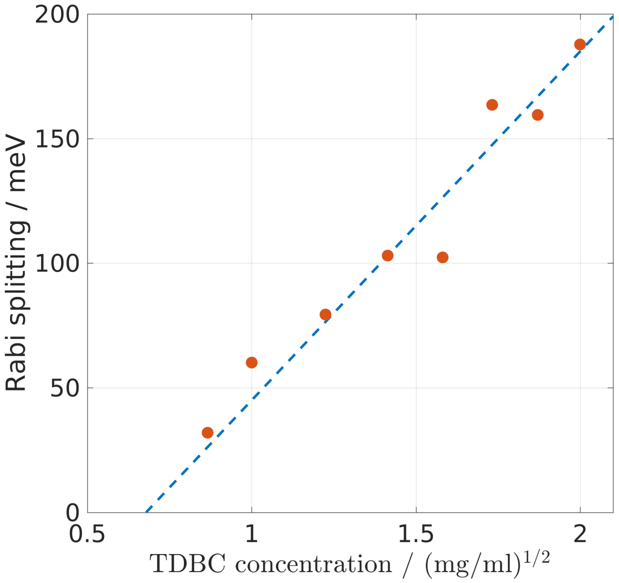

Supplementary Figure 4: **Polaritons with the organic component TDBC only.** Rabi splitting as a function of the square root of the TDBC concentration for a fixed cavity length, revealing a linear dependence when plotted against the square root of the dye concentration.

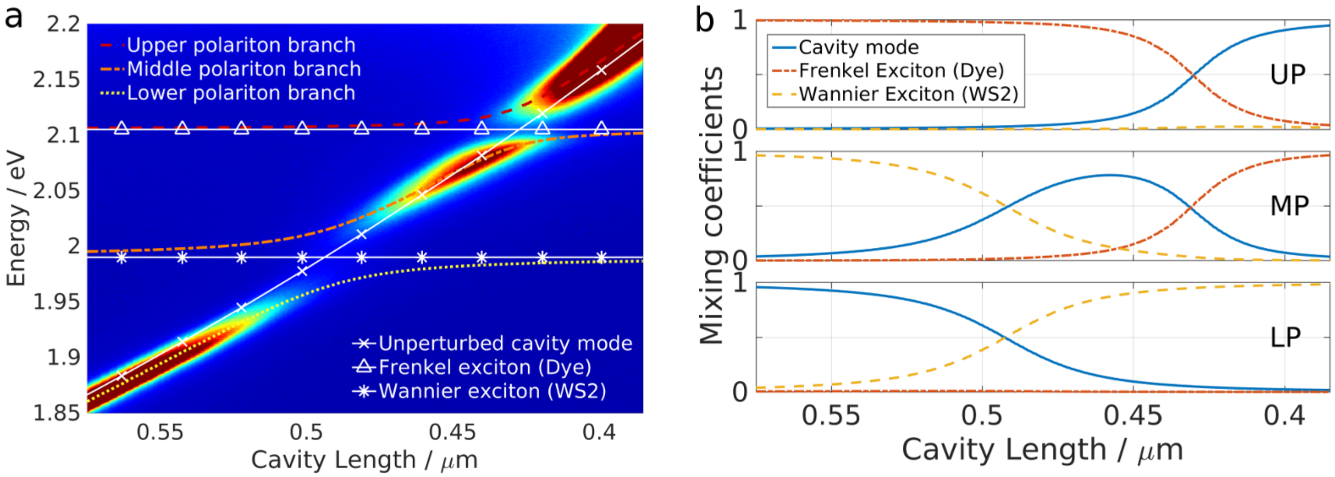

Supplementary Figure 5: **Hybrid polariton formation with lower concentration dye.** a) Successive transmission spectra as the cavity length is decreased from left to right, symbols and lines as in main text. b) Hopfield coefficients as presented in main text.

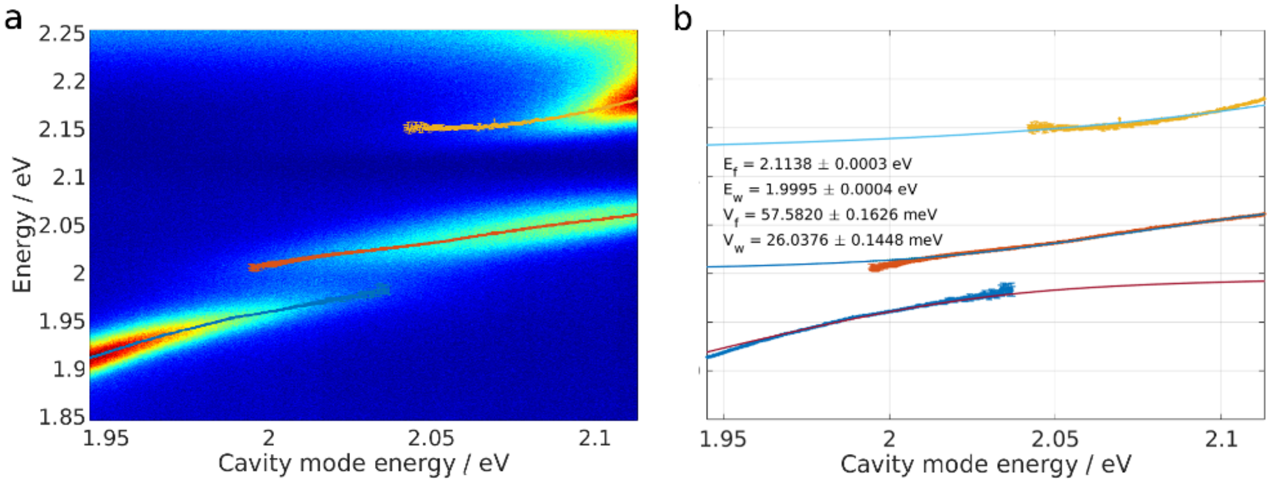

Supplementary Figure 6: **Peak and dispersion fitting** a) Fitted transmission peaks with error bars superimposed on raw transmission data for different cavity mode energies. Each peak is fitted with a Lorentzian profile. b) Dispersion as given by Eq. 2 fitted to the peak positions presented in a. The fit is performed after obtaining three separate equations for UP, MP and LP from diagonalisation of Eq. 2 and proceeding with a non-linear least squares algorithm with shared parameters E_{f}, E_{w}, V_{f} and V_{w}.

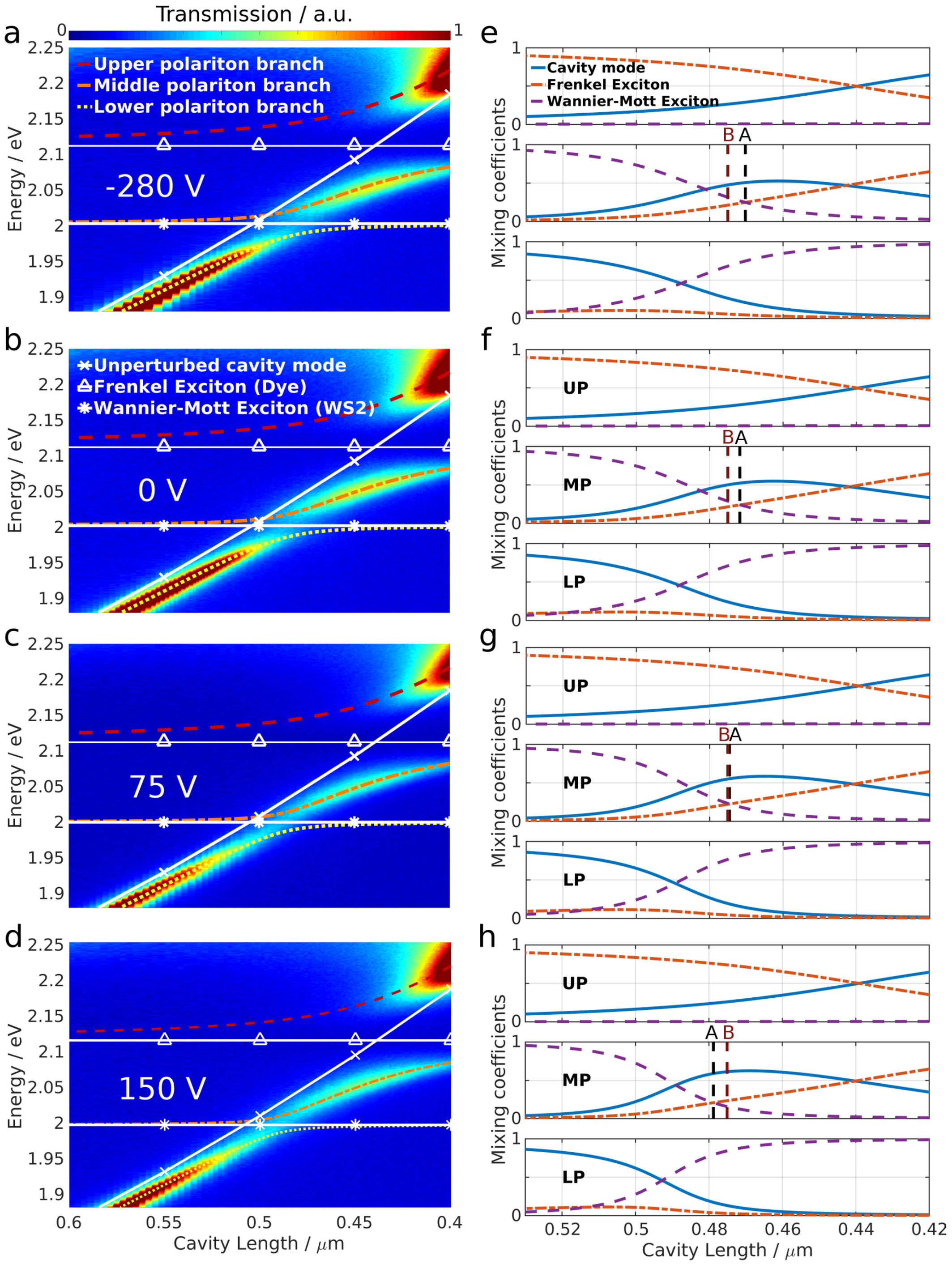

Supplementary Figure 7: **Electrical tuning of hybrid polaritons on a second WS_2 flake.** a-d) Successive transmission spectra of hybrid WS_2-TDBC microcavity for decreasing cavity length from left to right and different applied voltages of -280 V, 0 V, 75 V and 150 V for a, b, c and d respectively. The white, continuous lines correspond to the uncoupled energies of Frenkel-exciton (TDBC, triangles), Wannier-Mott exciton (WS_2, stars) and cavity mode (crosses). The dashed lines in colour show the dispersion for the coupled system consisting of the three polariton branches, lower polariton (LP, yellow, - -), middle polariton (MP, orange, – -) and upper polariton (UP, red, – –). e-h) Photonic (cavity mode, blue, continuous), Frenkel-excitonic (TDBC, red,– -) and Wannier-Mott-excitonic (WS_2, purple, - -) contribution to the three polariton branches LP, MP and UP for the dispersions plotted respectively above. Two points A (black dashed line) and B (red dashed line) mark cavity lengths at which: A) Frenkel- and Wannier-Mott-exciton contribution to the middle polariton branch is equal, B) the cavity length is $L = 0.475\,\mu\text{m}$.

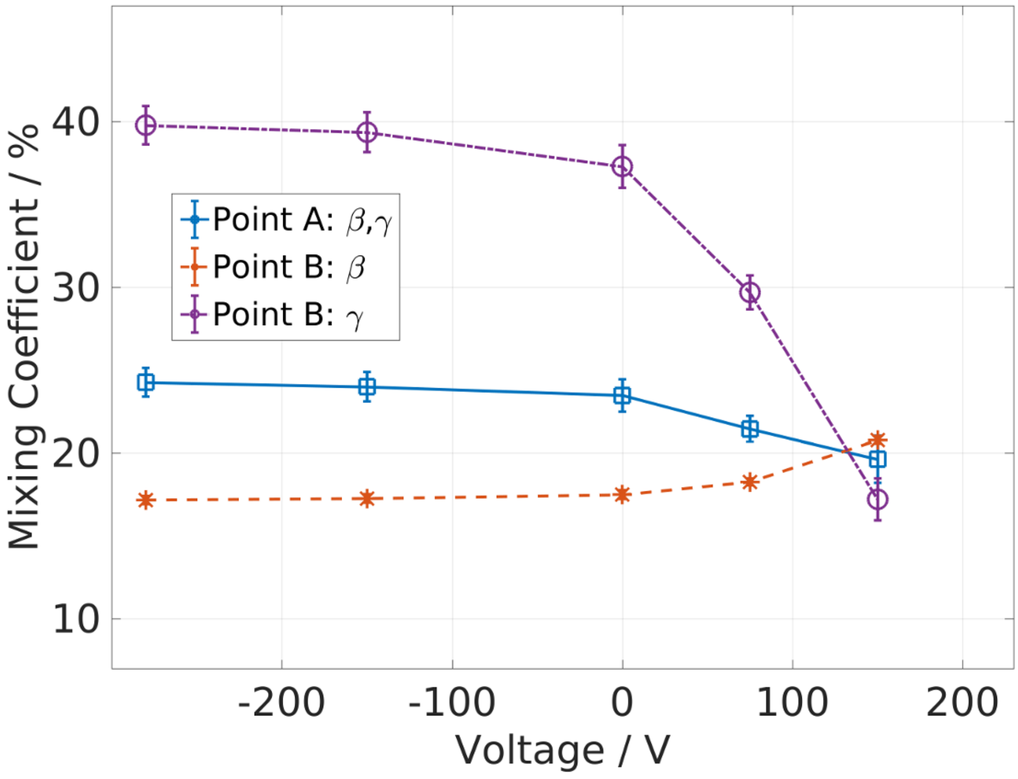

Supplementary Figure 8: **Electrical control over composition of polaritons for a second WS_2 flake.** Frenkel- and Wannier-Mott-exciton fraction β and γ in hybrid polariton state for different applied voltages (colour, symbols, left ordinate) and absorbance peak position of WS_2 outside the cavity (black, dashed, right ordinate). The exciton fractions are shown for two points, where point A corresponds to the cavity length at which $\beta = \gamma$ (maximal mixing) and point B to a fixed cavity length of $L = 0.475\,\mu m$.

Supplementary Note 1. Details of experimental setup

A. Sample preparation

In the following section we describe the two mirrors forming the cavity and the absorbing material within. more details on the measurements are given in the next section. The first mirror consists of 10 pair DBR of SiO_2, TiO_2 with refractive indices 1.45 and 2.05 respectively deposited on a 0.5 mm thick, flat Silica substrate. The stopband of this mirror is centered around $\lambda = 637$ nm and the reflectivity at that wavelength is 99.7%. The second, smaller mirror is produced by removing large areas of a flat silica substrate with a dicer to create a 200×300 µm^2 plinth made semi-reflective by thermally evaporating a 50 nm thick silver layer. To enable electrical control within the cavity, silver electrodes with a width and a spacing of 90 µm are thermally evaporated on top of the DBR. This process is facilitated by masking a region of the DBR with a laser processed foil defining the electrode shape. The thickness of these electrodes is similar to the thickness of the silver layer on the small opposing mirror, approximately 50 nm. The WS_2 flakes are grown as described in [S1] and transferred onto the dielectric mirror stack, which has a low refractive-index terminated layer to provide an anti-node of the electric field at the mirror surface and thus optimal coupling to the monolayer. This transfer is facilitated by spin-coating a thick layer of PMMA onto the as-grown WS_2 flakes on their native SiO_2 substrate. After etching the substrate away the PMMA stays connected with the WS_2 flakes and can be fished out and cut manually. The resulting PMMA section is then manually positioned over the DBR region holding the electrodes and baked at 100°C for one hour. After this the PMMA is removed by putting the sample into an acetone bath for about 15 min. The distribution of WS_2 flakes relative to the electrodes is random, the average size of a WS_2 flake being 80 µm, with 90% of the flakes overlapping partially with the silver electrodes (see Fig. 1). The results presented above were obtained from a flake which was not in contact with the electrodes, thus ensuring purely electrostatic tuning. The electrodes have a length of ≈ 1 cm and were connected to thin wires with conducting silver paint at the respective ends. A cavity mode in the region of these electrodes would be spectrally broader and the number of round-trips would be reduced due to the additional absorption of the second silver interface. For the data that we present in the manuscript the effect that such broadening would have can be estimated:
Given the cavity length of around 500 nm and the reflectivity of the silver mirror $R = 0.95$, we can equate an effective mode area $A = \frac{\pi L \lambda}{1-R} \approx 19.5\ \mu m^2$, as derived in [S2]. This area translates to a mode radius of $r \approx 2.5\ \mu$m. Since the region of the sample from which we obtained the results is more than 15 μm from the next silver electrode, the effect from the electrode on the cavity mode is negligible.

The organic dye was introduced on the opposing mirror by dissolving J-aggregated 1,1′-diethyl-3,3′-di(4-sulfobutyl)-5,5′,6,6′-tetrachlorobenzimidazolocarbocyanine (TDBC) in an aqueous solution with 5 weight percent gelatine. The solution was then spin coated onto the small silver mirror, giving a polymer, dye layer of approximately 300 nm. The j-aggregate exciton energy can be shifted in an electrical field through the Stark effect, but it requires field strengths of about 10^6 V/cm for a 20 nm shift (as determined through electroabsorption measurements - unpublished), whereas the field strengths that we obtained in our experiment were about 2.33×10^4 V/cm. Our data gives experimental evidence of this, since the Frenkel exciton energy remained unchanged (within the uncertainty of the Lorentzian lineshape fit) for any applied electric field.

B. Optical measurements

The small silver mirror is mounted on a three-dimensional piezo actuated stage, which makes electric positioning relative to the WS_2 flake possible. To initialise the cavity, white light from a light emitting diode is shone through the mirrors while reducing the separation. With the help of Fabry-Perot fringes visible for small mirror separations ($L < 20$ µm) both surfaces are made parallel within 150 µrad. Optical access to the sample is given by a standard ×10 objective lens and the collected light is focused on an Andor combined spectrograph/CCD for analysis. The electrodes were pairwise connected to a Keithley 2400 to apply voltages and monitor the current. Care was taken that for the datasets presented, the full voltage was applied and the respective current limits were not exceeded.

Supplementary Note 2. Components of hybrid polariton system

In the following we present a characterisation of the individual components of the hybrid system. Fig. 2a shows transmission spectra of a microcavity with only a WS_2 flake for different cavity lengths. As the cavity length is decreased from left to right, the cavity mode energy increases and traverses the exciton energy. As both components strongly couple to each other, an avoided level crossing with characteristic Rabi splitting is visible. The coloured,

dashed lines show the dispersion of the lower and upper polariton branch. Fig. 2b shows the photonic and excitonic fractions of the resulting polariton states, which are known as the Hopfield coefficients, as introduced in the main text. We have published a study on the properties of this open cavity polariton system [S3].

A strongly coupled system on the basis of organic dyes is well known in the literature [S4, S5]. Fig. 4 shows the Rabi splitting for a fixed cavity length as a function of the concentration of the dye TDBC. When plotted as a function of the square root of the concentration the linear relationship is visible. By varying the concentration of TDBC in the aqueous solution it is possible to optimise the hybridisation between WS_2 and organic excitons.

Fig. 5a shows transmission spectra of a hybrid microcavity system with a lower concentrated TDBC layer compared to the data presented before in the manuscript. Symbols and lines are defined as previously in the main text. As the strength of the interaction between dye and cavity mode is reduced, the photonic fraction in the middle polariton branch is increased and the hybridisation between both excitons only occurs marginally ($\beta^2 = \gamma^2 \approx 10\%$ for a cavity length of $L = 0.455\,\mu\text{m}$). By variation of the concentration of the dye it is thus possible to control the composition of the polariton branches, in particular the middle polariton branch. When relying on the photonic part of a polariton state for detection, a reduced overlap between both excitons is desirably - hence the choice of concentration in the main text. For applications, where the exciton hybridisation is the only important parameter, a larger dye concentration might be desirable.

Supplementary Note 3. Electrical control of WS_2 absorption profile

The occurence of trion (or charged exciton) states in atomically flat TMDCs has been reported previously [S6, S7]. By applying an electric field to deplete a region of the material from electrons, it is possible to change the Fermi level and thus the spectral weight and position of both the neutral exciton (X^0) and charged exciton (X^-) states [S6, S8–S10]. Such electrical control can thus be used to vary the absorption profile. Fig. 3 shows the absorbance of the WS_2 for different applied voltages. As the Fermi level is raised for increasing positive biases the spectral weight of the absorption profile shifts towards the charged exciton X^-.

As Fig. 2(a) in Ref. [S9] shows, the trion can be quenched by applying a negative gate voltage. The zero bias level of weight in neutral and charged exciton is dependent on the intrinsic doping of the WS_2 flake, which is thought to be a result of the growth and transfer method. On our CVD grown samples we see variations of X^- contribution on one flake and even larger variations when comparing multiple flakes. In general the X/X^- ratio found for our sample is similar to bias values between -30 V and 0 V in Fig. 2(a) in Ref. [S9]. To demonstrate the strong coupling to a cavity mode we chose a region with minimal trion contribution (as visible in the absorption lineshape in Fig. 1 d) for negative applied voltages). The reasoning behind this was, that with more trionic contribution the polariton linewidth increases, the asymmetry between upper and lower polariton branch increases and the splitting is less obvious.

Supplementary Note 4. Fitting procedure and error propagation

Each transmission data set is aquired and analysed by the following procedure: The sequence of transmission spectra is aquired by sweeping the voltage of the piezo microactuator, varying the cavity length. The actual cavity length for each frame is obtained by fitting a Lorentzian profile to the unperturbed cavity mode for either the same longitudinal mode (with index $q = 4$ and for energies below E =1.85 meV or the the next one (with index $q + 1 = 5$, if the $q = 4$ mode has energies above E =1.85 meV). The value of q can be obtained for each dataset from the free spectral range, which then allows the absolute cavity length to be expressed as $L_{cav} = \frac{qhc}{2E}$, where E is the mode energy. Each frame is fitted with Lorentzian lineshape peaks to obtain the position of the individual polariton branches (Fig. 6a). The analytic form of the three equations for UP, MP and LP is found by diagonalising Eq. 2. The three expressions are simultanuously fitted to the obtained peaks with a nonlinear least squares algorithm (Fig. 6b). In a first round, the four parameters (E_f, E_w, V_f, V_w) are shared in the fitting procedure and obtained as parameters after the fit. In a second round the parameters governing the position and the interaction strength of the dye (E_f, V_f) are fixed to a common value obtained by averaging their respective values from the first round. The covariance matrix M_cov is obtained for each fit. Through diagonalisation of Eq. 2 we obtain the eigenvectors, whose components represent the mixing coefficients after proper normalisation. The respective values for point A and B of these values is directly obtained by plugging in the parameters found above. The uncertainty for each of the coefficients is obtained by constructing the Jacobian $J^f_{ij} = \frac{\partial f_i}{\partial x_j}$, where f_i is the respective expression for the polariton fraction and x_j is one of the parameters found above. The uncertainties u can now be calculated from $u = \sqrt{\text{diag}(\text{JM}_\text{cov}\text{J}^\text{t})}$.

Supplementary Note 5. Electrical control on different WS_2 flake

Complementary to the data set shown in the main text, we include another dataset showing the electrical control of the hybridisation. Fig. 7a-d show transmission spectra at a different point on the sample for different applied voltages. The symbols and lines are defined as in the main text and the figure caption. Fig. 7e-h show the photonic (blue, continuous), Wannier-Mott (purple, dashed) and Frenkel-excitonic (red,dashed) fractions of the three polariton branches corresponding to the dispersion shown to the left in a-d.

Fig. 8 presents the summary of the electrically controlled hybridisation analogous to Fig. 3 in the main text for the second dataset.

Supplementary references

[S1] Rong, Y. *et al.* Controlling sulphur precursor addition for large single crystal domains of WS_2. *Nanoscale* **6**, 12096–12103 (2014).
[S2] Ujihara, K. Spontaneous Emission and the Concept of Effective Area in a Very Short Optical Cavity with Plane-Parallel Dielectric Mirrors. *Japanese Journal of Applied Physics* **30**, L901–L903 (1991).
[S3] Flatten, L. C. *et al.* Room-temperature exciton-polaritons with two-dimensional WS2. *Scientific Reports* **6**, 33134 (2016).
[S4] Coles, D. M., Grant, R. T., Lidzey, D. G., Clark, C. & Lagoudakis, P. G. Imaging the polariton relaxation bottleneck in strongly coupled organic semiconductor microcavities. *Physical Review B* **88**, 121303 (2013).
[S5] Coles, D. M. *et al.* Polariton-mediated energy transfer between organic dyes in a strongly coupled optical microcavity. *Nature Materials* **13**, 712–719 (2014).
[S6] Chernikov, A. *et al.* Electrical Tuning of Exciton Binding Energies in Monolayer WS_2. *Phys. Rev. Lett.* **115**, 126802 (2015).
[S7] Singh, A. *et al.* Trion formation dynamics in monolayer transition metal dichalcogenides. *Physical Review B* **93**, 041401 (2016).
[S8] Ross, J. S. *et al.* Electrical control of neutral and charged excitons in a monolayer semiconductor. *Nature Communications* **4**, 1474 (2013).
[S9] Zhu, B., Chen, X. & Cui, X. Exciton Binding Energy of Monolayer WS2. *Scientific Reports* **5**, 9218 (2015).
[S10] Sidler, M. *et al.* Fermi polaron-polaritons in charge-tunable atomically thin semiconductors. *Nature Physics* (2016).
